# Effects of Memantine on okadaic acid induced neurotoxicity at the systemic and molecular level in rats

**DOI:** 10.1002/alz.087916

**Published:** 2025-01-03

**Authors:** Khatuna Rusadze, MARIAM CHIGHLADZE, Manana Dashniani, Maia Burjanadze, Nino Chkhikvishvili, Gela Beselia

**Affiliations:** ^1^ Akaki Tsereteli State University, Kutaisi Georgia; ^2^ Kutaisi University, Kutaisi Georgia; ^3^ Ivane Beritashvili Center of Experimental Biomedicine, Tbilisi Georgia

## Abstract

**Background:**

Alzheimer’s disease (AD) is a neurodegenerative disease that causes progressive cognitive decline over age 65. Individuals suffering from this disease suffer memory loss, and histological examination of the brains. Okadaic acid (OA), is a potent and selective inhibitor of protein phosphatases 1 and 2A. Intracerebroventricular (ICV) administration of OA can produce an Alzheimer‐like hyperphosphorylation of tau protein. Therefore, it was suggested that intracerebral injection of OA can affect the activity of neurotransmitters and their receptors in brain areas. Central cholinergic and glutamatergic activity together are known to be crucial for the acquisition and consolidation performance of a variety of learned behaviors. The present study was designed to investigate the effects of okadaic acid (OA) intracerebroventricular (ICV) injection on memory function and expression level of α7 subunit of nicotinic acetylcholine receptor (nAChR) and NR2B subunit of NMDA glutamate receptors in the hippocampus, as well as effect of the antidementic drug memantine on OA induced changes at systemic and molecular level in rats.

**Method:**

OA was dissolved in artificial cerebrospinal fluid (aCSF) and injected ICV 200 ng/10 μl. Vehicle control received 10 μl of aCSF ICV. Control and OA injected rats were divided into 2 subgroups: treated i.p. with saline or memantine (5 mg/kg daily for 13 days starting from the day of OA injection). Rats were trained in the dual‐solution plus‐maze task that can be solved by using place or response strategies. The Western immunoblotting was used to determine relative amount of hippocampal receptors protein levels.

**Result:**

Obtained data revealed that OA ICV injected rats were severely impaired at acquiring the place version of the maze accompanied by significant decline in hippocampal α7 subunit of nACh receptors protein levels. Memantine treatment can prevent OA induced impairment of hippocampal‐dependent spatial memory and accompanied by modulation of the expression level of α7 subunit of nACh and NR2B subunit of NMDA receptors in the hippocampus.

**Conclusion:**

Thus, our results support the presumption that α7 nACh receptors may play an important role in the cognitive enhancer effects of memantine and emphasize the role of cholinergic‐glutamatergic interactions in memory.

